# Physical (in)activity and screen-based media use of adolescents with juvenile idiopathic arthritis over time - data from a German inception cohort

**DOI:** 10.1186/s12969-024-01027-6

**Published:** 2024-10-21

**Authors:** Florian Milatz, Malthe Jessen Pedersen, Jens Klotsche, Ina Liedmann, Martina Niewerth, Anton Hospach, Gerd Horneff, Ariane Klein, Frank Weller-Heinemann, Ivan Foeldvari, Tilmann Kallinich, Johannes-Peter Haas, Daniel Windschall, Frank Dressler, Dirk Foell, Jasmin B. Kuemmerle-Deschner, Kirsten Minden

**Affiliations:** 1 Programme Area Epidemiology and Health Services Research, Deutsches Rheuma-Forschungszentrum Berlin, a Leibniz Institute, Charitéplatz 1, 10117 Berlin, Germany; 2 Institute of Social Medicine, Epidemiology and Health Economics, Charité - Universitätsmedizin Berlin, corporate member of Freie Universität Berlin and Humboldt-Universität zu Berlin, Berlin, Germany; 3 Partner site Berlin, German Center for Child and Adolescent Health (DZKJ), Berlin, Germany; 4 Department of Public Health, Aarhus University, Aarhus, Denmark; 5 Department of Paediatrics, Klinikum Stuttgart, Olgahospital, Stuttgart, Germany; 6 Department of General Paediatrics, Asklepios Clinic Sankt Augustin, Sankt Augustin, Germany; 7 Department of Paediatric and Adolescents Medicine, University Hospital of Cologne, Cologne, Germany; 8 Department of Pediatrics and Adolescent Medicine, Pediatric Rheumatology, Eltern-Kind-Zentrum Prof. Hess, Klinikum Bremen Mitte, Bremen, Germany; 9 Hamburg Centre for Paediatric and Adolescent Rheumatology, Schön Klinik Hamburg Eilbek, Hamburg, Germany; 10 Department of Paediatric Respiratory Medicine, Immunology and Critical Care Medicine, Charité – Universitätsmedizin Berlin, Corporate Member of Freie Universität Berlin and Humboldt-Universität zu Berlin, Berlin, Germany; 11 Systems Rheumatology, Deutsches Rheuma-Forschungszentrum Berlin, Berlin, Germany; 12 German Centre for Paediatric and Adolescent Rheumatology, Garmisch-Partenkirchen, Germany; 13 Clinic of Paediatric and Adolescent Rheumatology, Northwest German Centre for Rheumatology, St. Josef-Stift Sendenhorst, Sendenhorst, Germany; 14 Medizinische Fakultät, Universität Halle-Wittenberg, Halle, Germany; 15 Department of Paediatric Pneumology, Allergology and Neonatology, Children’s Hospital, Hannover Medical School, Hannover, Germany; 16 Department of Paediatric Rheumatology and Immunology, University Hospital Muenster, Muenster, Germany; 17 Division of Paediatric Rheumatology and Autoinflammation Reference Centre Tuebingen (arcT), Department of Paediatrics, University Hospital Tuebingen, Member of ERN-RITA, Tuebingen, Germany

**Keywords:** Physical activity, Screen time, Media use, Juvenile idiopathic arthritis, Adolescents, Sedentary behavior

## Abstract

**Background:**

Regular physical activity (PA) has been proven to help prevent non-communicable diseases and is beneficial for disease management in chronically ill populations. Physical inactivity and recreational screen-based media (SBM) use are related to poor health outcomes and common among youth. This study aimed to (1) investigate PA levels and recreational SBM use of adolescents with JIA over time and (2) compare these behaviours with those of their peers.

**Methods:**

Data from JIA patients and their peers enrolled in the inception cohort study ICON at 11 German centers were analyzed. Individuals aged 13 and over were followed prospectively with questionnaires concerning PA level, recreational SBM use, and health-related quality of life (HRQoL) at a two-year interval. Group by time interactions were analyzed using linear mixed models.

**Results:**

Data of 214 patients (mean age at first documentation 14.4 ± 0.9 years, female 63%) and 141 peers could be considered. At first documentation, patients were less physically active compared to their peers (*p* < 0.001). In contrast to their peers, patients’ PA levels increased over time (OR 3.69; 95% CI: 1.01–13.50, *p* = 0.048). Mean screen time did not differ significantly between patients and peers (first documentation: 3.5 h vs. 3.0 h, *p* = 0.556; follow-up: 3.6 h vs. 3.3 h, *p* = 0. 969). During the observation period, male patients reported higher PA levels than female patients, but also higher screen time levels. While low socioeconomic status (SES) (OR 14.40; 95%-CI: 2.84–73.15) and higher cJADAS-10 score (OR 1.31; 95%-CI: 1.03–1.66) increased the likelihood for high SBM use (≥ 4.5 h/d), higher PedsQL psychosocial health score (OR 0.93; 95%-CI: 0.88–0.99) was associated with a decreased likelihood.

**Conclusions:**

Adolescents with JIA become more physically active over the disease course and achieve comparable levels of PA and recreational screen time to their peers. However, the vast majority appear to be insufficiently physically active. Future interventions to promote healthy lifestyles should include gender and SES as important determinants to reach most vulnerable groups.

**Supplementary Information:**

The online version contains supplementary material available at 10.1186/s12969-024-01027-6.

## Background

Juvenile idiopathic arthritis (JIA) is the most common immune-mediated inflammatory rheumatic disease affecting the pediatric population. JIA encompasses six defined JIA categories occurring before the age of 16 and characterized by an overproduction of inflammatory cytokines, leading to a chronic inflammatory state [1]. With disease onset, children and adolescents are faced with a challenging new way of life, often with a cluster of disabling symptoms known as “sickness behaviour”, including regular painful crises and fatigue [2, 3]. These can profoundly affect physical, mental, social, and functional aspects of life, drastically reducing health-related quality of life (HRQoL) [4–6]. Patients with JIA may exhibit lower physical fitness [7] and activity [8, 9], more fragmented sleep [10], and alterations in body composition such as higher adiposity than healthy controls [11].

A relatively sedentary lifestyle including insufficient physical activity (PA) and high levels of recreational screen-based media (SBM) use is associated with an increased burden of many non-communicable diseases [12, 13]. As lifestyle habits developed during adolescence tend to be maintained throughout life, it is therefore essential to promote healthy lifestyle habits among this population [14].

High screen time has been shown to be associated with social isolation, poor relationships with friends or family, depressive symptoms, and negative effects on mood as well as cognitive and emotional development, leading to poorer academic performance and HRQoL [15–18]. Insufficient PA, defined as failing to meet the WHO recommendation of at least 60 min moderate to vigorous PA on average per day predicts a wide range of short- and long-term health problems that are detrimental to well-being [19].

Recent research indicates that adolescents with chronic diseases are equally or even more likely to be physically inactive and have high levels of sedentary screen time compared to their healthy peers [20–23]. Both behaviors have been associated with poor health-related outcomes in autoimmune rheumatic diseases [24], potentially aggravating common features such as weakness, atrophy and dysfunction of muscles, chronic pain and fatigue, bone loss, dyslipidemia, arterial hypertension, and insulin resistance [25]. Moreover, young patients with JIA display a number of symptoms that could potentially be mitigated by increasing PA levels [25].

Despite these facts and knowledge about beneficial effects of sufficient PA on the balance between pro- and anti-inflammatory responses [26], there are to date only remarkably few studies investigating sedentary activities in patients with JIA. These have either examined these activities only in younger children or only based on very small sample sizes [21, 27]. In addition, none of these previous studies prospectively investigated recreational SBM use and PA behaviour during the disease course and with consideration of HRQoL. Given these research gaps, the current study aimed to examine daily time spent on recreational SBM, levels of weekly PA, and HRQoL in adolescents with JIA over time and examine potential differences compared to their peers.

## Methods

### Study design

Data from the German multicenter Inception Cohort of Newly diagnosed patients with JIA (ICON) were used. ICON was a prospective observational study aiming to follow patients with recent onset of JIA according to the International League of Associations for Rheumatology (ILAR) classification criteria [1] for at least 10 years. Eleven of the largest pediatric rheumatology centers in Germany recruited patients from 2010 onwards and documented clinical characteristics and treatments by a standardized physician questionnaire several times a year until the end of 2021. At the same time as the rheumatologists, patients ≥ 13 years reported on their state of health. Healthy peers were recruited with the help of patients and/or their parents who asked friends of equal age and gender to serve as peers. These young people completed a questionnaire at home once a year. Further details on the ICON cohort study, containing sociodemographic and clinical characteristics as well as treatments are provided by Sengler et al. [28].

SBM and PA evaluations in JIA patients and peers took place every 2 years. The 24-month follow-up (FU) was chosen as the study endpoint. Data cut-off for this analysis was 31 Dec 2019. Inclusion criteria for the analyses in the present study were as follows: (1) diagnosis of JIA according to the ILAR criteria [29], (2) age at documentation of at least 13 years, and (3) availability of two completed evaluations regarding SBM or PA per patient and per peer. The study protocol was approved by the ethics committee of the Charité - Universitätsmedizin Berlin [EA1/056/10].

### Clinical data

Sociodemographic and clinical information such as age, gender, weight, height, disease onset, date of diagnosis, JIA category, drug treatment, number of active joints, and global assessment of disease activity was provided by the treating pediatric rheumatologist. Physician`s global assessment of disease activity was assessed using a numerical rating scale (NRS) (scale from 0 = no disease activity to 10 = very severe disease activity). Body mass index (BMI) was calculated as the weight in kilograms divided by the height in meters squared according to age- and gender-specific percentiles used in the German reference system [30, 31].

Patient-reported outcomes included the assessment of overall well-being and pain using NRS ranging from 0 to 10 (0 representing the best possible outcomes). Patients’ functional abilities were assessed using the German version of the C-HAQ [32].

Disease activity was evaluated by the clinical Juvenile Arthritis Disease Activity Score (cJADAS-10) [33]. The cJADAS-10 (range 0–30, 30 = very severe disease activity) is calculated with the physician’s global assessment of disease activity, the patient’s global assessment of overall well-being and the number of active joints (up to a maximum of 10).

HRQoL was reported using the German versions of the Pediatric Quality of Life Inventory generic core scales (PedsQL™ 4.0) [34, 35]. Consisting of four subscales (physical functioning, emotional functioning, social functioning and school functioning), the PedsQL™ can be combined into psychosocial and physical health composite scales, as well as the PedsQL™ total score. PedsQL™ scores range from 0 being the worst to 100 being the best possible HRQoL.

To measure adolescents’ socioeconomic status (SES) an established German multidimensional aggregated index was used. This index was evaluated and adapted from a representative German population sample of 17,641 study participants aged up to 17 years [36]. As the parental work status was not ascertained in the ICON study, the calculation of this index was modified to be based only on parental education level (including school education and vocational training) and net household income according to the method used by Listing et al. [37]. The lower and upper quintiles of the sum of the education and income scores (6.55, 12.1) were used as cut-off points to define low, moderate, and high SES.

### SBM use and PA

Patients and their peers were asked how often they are physically active in their leisure time in such a way they sweat or breathe hard (e.g. by sports or bicycling). Possible answers were: ‘about every day’, ‘about 3–5 times a week’, ‘about once or twice a week’, ‘about once or twice a month’, or ‘never’. Because the last two categories were rare, they were summarized as “no regular physical activity” and used as reference in the main analyses.

Types of SBM were assessed using a questionnaire, which asked for the overall amount of daily time spent with different screen media (television/videos, computer/Internet, and gaming consoles). These questions explicitly referred to recreational screen time and not to the use of SBM for educational purposes. According to a large population-based representative study [38], an index for screen time was formed: for television/videos, computer/internet, and gaming consoles, individual answers were scored with 0 (‘not at all’), 0.5 (‘about half an hour’), 1.5 (‘about 1–2 h’), 3.5 (‘about 3–4 h’), and 5 (‘more than 4 h’) and summed up across these media. The total screen time index was only computed for adolescents with valid answers for all three media. Because the distribution of the SBM index was skewed and to harmonize the analyses, SBM was classified into four groups: below 2 h per day (used as the reference category in the analyses), 2–<3 h/day, 3–<4.5 h/day, and ≥ 4.5 h/day. Similar SBM items have shown good retest reliability and good criterion-related validity in other applications with adolescent samples [39, 40].

### Statistical analysis

Categorical variables were reported by numbers and percentages, whereas continuous variables were reported by means and standard deviations. Ordinal logistic regression analyses (for categorical variables) and linear regression analyses (for continuously distributed variables) were performed to estimate differences between males and females (adjusted for SES) and patients and peers (adjusted for SES and gender). An omnibus test was used in regression analyses in order to test whether JIA categories significantly explain the variance in total screen time and PA.

Multinomial logistic regression was performed to estimate the relationship between SBM use/PA levels/HRQoL and clinical parameters. SES and gender were included as potential covariates, as these variables are commonly associated with PA and SBM use [41]. As the rate of missing values across the parameters was low (< 15%), no imputation of data was performed. The association between total screen time, PA level, and clinical/sociodemographic characteristics is described by odds ratios (ORs) with 95% confidence intervals (CIs). Linear mixed models were used to compare the patient and peer groups at different time points.

All p-values less than 0.05 were considered to be statistically significant. Statistical analyses were performed using IBM SPSS Statistics version 25 (IBM Corp., Armonk, NY, USA) and SAS 9.3 (SAS Institute, Cary, NC, USA).

## Results

### Sociodemographic and clinical information

In total, data on SBM use or PA were available for 214 adolescents with JIA and 141 peers. Mean observation time was 2.1 ± 0.3 years. The most common JIA category was rheumatoid factor negative polyarthritis (28.1%) followed by enthesitis-related arthritis (21.0%). During the observation period of 24 months, the proportion of patients treated with DMARDs increased from 52.2 to 58.8%, mainly due to an increase in patients treated with biologics (from 21.4 to 38.2%). With more frequent treatment with biologics, the proportion of patients with inactive disease and unrestricted functional ability increased over time. More details on patients’ characteristics are presented in Table 1.

**Table 1:** Characteristics of JIA patients and their peers at first documentation and 2-year follow-up

Variables	First documentation		2-year follow-up	
	JIA	Peers	JIA	Peers
No. of patients	214	141	214	141
Age, years	14.4 ± 0.9	14.5 ± 1.0	16.4 ± 0.9	16.5 ± 1.0
BMI, kg/m²	21.1 ± 3.5	20.9 ± 3.4	21.6 ± 3.9	21.4 ± 3.3
Female, no. (%)	135 (63.1)	77 (54.6)	135 (63.1)	77 (54.6)
Disease duration, years	2.6 ± 2.0		4.7 ± 2.0	
JIA category, no. (%)				
RF-positive polyarthritis	10 (4.8)		10 (4.8)	
RF-negative polyarthritis	59 (28.1)		59 (28.1)	
Systemic JIA	6 (2.9)		6 (2.9)	
Persistent oligoarthritis	37 (17.6)		37 (17.6)	
Extended oligoarthritis	18 (8.6)		18 (8.6)	
Psoriatic arthritis	18 (8.6)		18 (8.6)	
Enthesitis-related arthritis	44 (21.0)		44 (21.0)	
Unclassified JIA	18 (8.6)		18 (8.6)	
cJADAS-10, mean (SD)	6.1 ± 6.0		3.1 ± 3.8	
PGA score (NRS 0–10), mean (SD)	1.85 ± 2.3		0.7 ± 1.3	
Inactive disease, no. (%)	63 (35.4)		93 (56.4)	
No. of active joints	2.1 ± 4.2		0.7 ± 1.8	
**Patient-reported data**				
C-HAQ total score, mean (SD)	0.3 ± 0.5		0.2 ± 3.9	
No functional limitations*, no. (%)	104 (50.0)		134 (65.0)	
Overall well-being (NRS 0–10), mean (SD)	2.1 ± 2.3	1.0 ± 1.4	1.5 ± 2.0	1.0 ± 1.2
Pain intensity (NRS 0–10), mean (SD)	2.1 ± 2.5	1.0 ± 1.5	1.4 ± 1.9	1.2 ± 1.8
PedsQL total score, mean (SD)	84.5 ± 16.3	91.4 ± 7.8	89.6 ± 13.2	91.2 ± 8.5
PedsQL physical health, mean (SD)	80.7 ± 21.7	93.5 ± 9.3	87.9 ± 17.2	93.0 ± 8.9
PedsQL psychosocial health, mean (SD)	86.5 ± 15.4	90.3 ± 8.7	90.4 ± 12.7	90.3 ± 9.7
Low/medium SES	171 (81.4)	90 (63.8)	171 (81,4)	90 (63.8)
High SES	39 (18.6)	51 (36.2)	39 (18.6)	51 (36.2)
**Current treatment**, **no. (%)**				
Any DMARD	95 (52.2)		97 (58.8)	
Any conventional synthetic DMARD	75 (41.2)		61 (37.0)	
Any biologic DMARD	39 (21.4)		63 (38.2)	
NSAIDs	79 (43.4)		31 (18.8)	
Systemic GCs	30 (16.5)		11 (6.7)	

*JIA* juvenile idiopathic arthritis; *RF* rheumatoid factor; *cJADAS-10* 10-joint clinical Juvenile Arthritis Disease Activity Score; *PGA* physician’s global assessment; *ESR* erythrocyte sedimentation rate; *C-HAQ* Childhood Health Assessment Questionnaire; *SES* socioeconomic status; *GC* glucocorticoid; *DMARD* disease-modifying antirheumatic drug

^Φ^Defined by a PGA score of zero

*Defined by a C-HAQ score of zero

## Associations between PA level, SBM use and patients’ characteristics

As shown in Table 2, female patients reported shorter total screen time than male patients both at first documentation (3.2 h vs. 4.2 h, *p* = 0.008) and at follow-up (3.2 h vs. 4.4 h, *p* = 0.003). They were less likely to consume more than 4 h of SBM per day than male patients (first documentation: 24.0% vs. 39.4%, *p* = 0.023, follow-up: 26.7% vs. 42.0%, *p* = 0.028).

**Table 2:** Physical activity and recreational screen time among adolescent girls and boys, adjusted for SES

First documentation	Girls			Boys		
	JIA	Peers	*p-value*	JIA	Peers	*p-value*
**Physical activity level**			**< 0.001**			0.830
Nearly every day, no. (%)	15 (11.3)	12 (16.2)		25 (31.6)	18 (27.7)	
3–5 times/week, no. (%)	29 (21.8)	31 (41.9)		20 (25.3)	18 (27.7)	
1–2 times/week, no. (%)	58 (43.6)	28 (37.8)		23 (29.1)	26 (40.0)	
1–2 times/month, no. (%)	19 (14.3)	3 (4.1)		4 (5.1)	3 (4.6)	
Never, no. (%)	12 (9.0)	0 (0)		7 (8.9)	0 (0)	
**Screen time level**			0.723			0.435
< 2h/day, no. (%)	32 (24.8)	14 (18.9)		10 (14.1)	11 (17.7)	
2-2.5h/day, no. (%)	37 (28.7)	30 (40.5)		15 (21.1)	13 (21.0)	
3-4h/day, no (%)	29 (22.5)	24 (32.4)		18 (25.4)	16 (25.8)	
> 4h/day, no. (%)	31 (24.0)	6 (8.1)		28 (39.4)	22 (35.5)	
Total screen time^¥^, h/day	3.2 ± 2.5	2.5 ± 1.2	0.351	4.2 ± 2.6	3.7 ± 2.4	0.870
**2-year follow-up**	**girls**			**boys**		
	**JIA**	**Peers**	***p-value***	**JIA**	**Peers**	***p-value***
**Physical activity level**			0.270			0.200
Nearly every day, no. (%)	18 (13.6)	8 (10.5)		13 (16.9)	16 (25.0)	
3–5 times/week, no. (%)	35 (26.5)	30 (39.5)		30 (39.0)	27 (42.2)	
1–2 times/week, no. (%)	55 (41.7)	28 (36.8)		25 (32.5)	18 (28.1)	
1–2 times/month, no. (%)	14 (10.6)	7 (9.2)		6 (7.8)	2 (3.1)	
Never, no. (%)	10 (7.6)	3 (3.9)		3 (3.9)	1 (1.6)	
**Screen time level**			0.484			0.789
< 2h/day, no. (%)	37 (28.2)	16 (22.2)		12 (17.4)	6 (10.0)	
2-2.5h/day, no. (%)	28 (21.4)	19 (26.4)		7 (10.1)	19 (31.7)	
3-4h/day, no (%)	31 (23.7)	26 (36.1)		21 (30.4)	17 (28.3)	
> 4h/day, no. (%)	35 (26.7)	11 (15.3)		29 (42.0)	18 (30.0)	
Total screen time^¥^, h/day	3.2 ± 2.3	3.1 ± 2.3	0.660	4.4 ± 2.6	3.6 ± 2.1	0.630

Male patients displayed higher PA levels than female patients at both first documentation (*p* < 0.001) and follow-up (*p* = 0.038), however, differences slightly decreased throughout the observation period (Table 2).

**Fig. 1:**
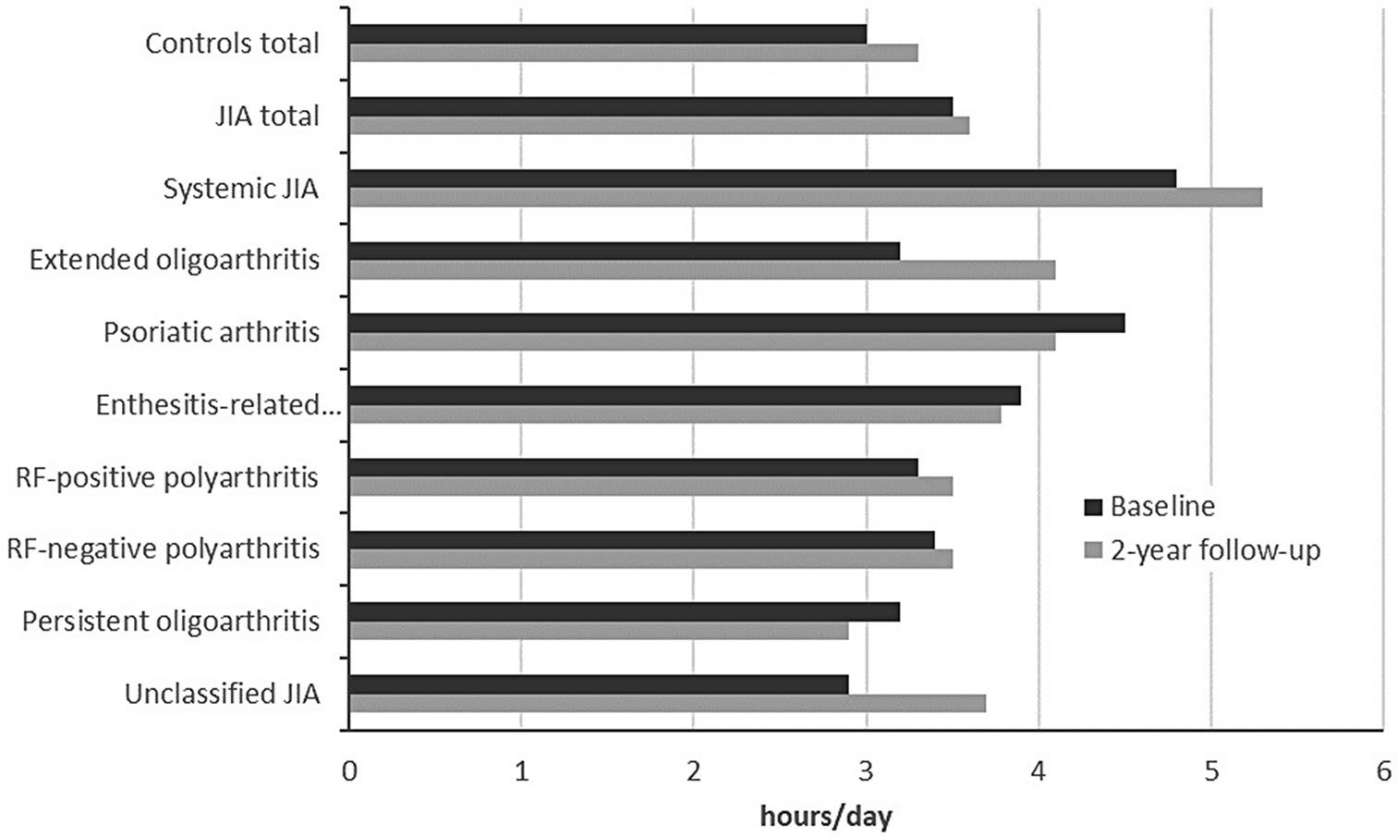
Frequency of physical activity within JIA categories at 2-year follow-up

The proportion of patients reporting to be physically active at most twice per month (“no regular PA”) was highest among patients with psoriatic arthritis and polyarthritis (Fig. 1). The overall level of PA did not differ significantly between JIA categories neither at first documentation nor at follow-up (results not shown).

**Fig. 2:**
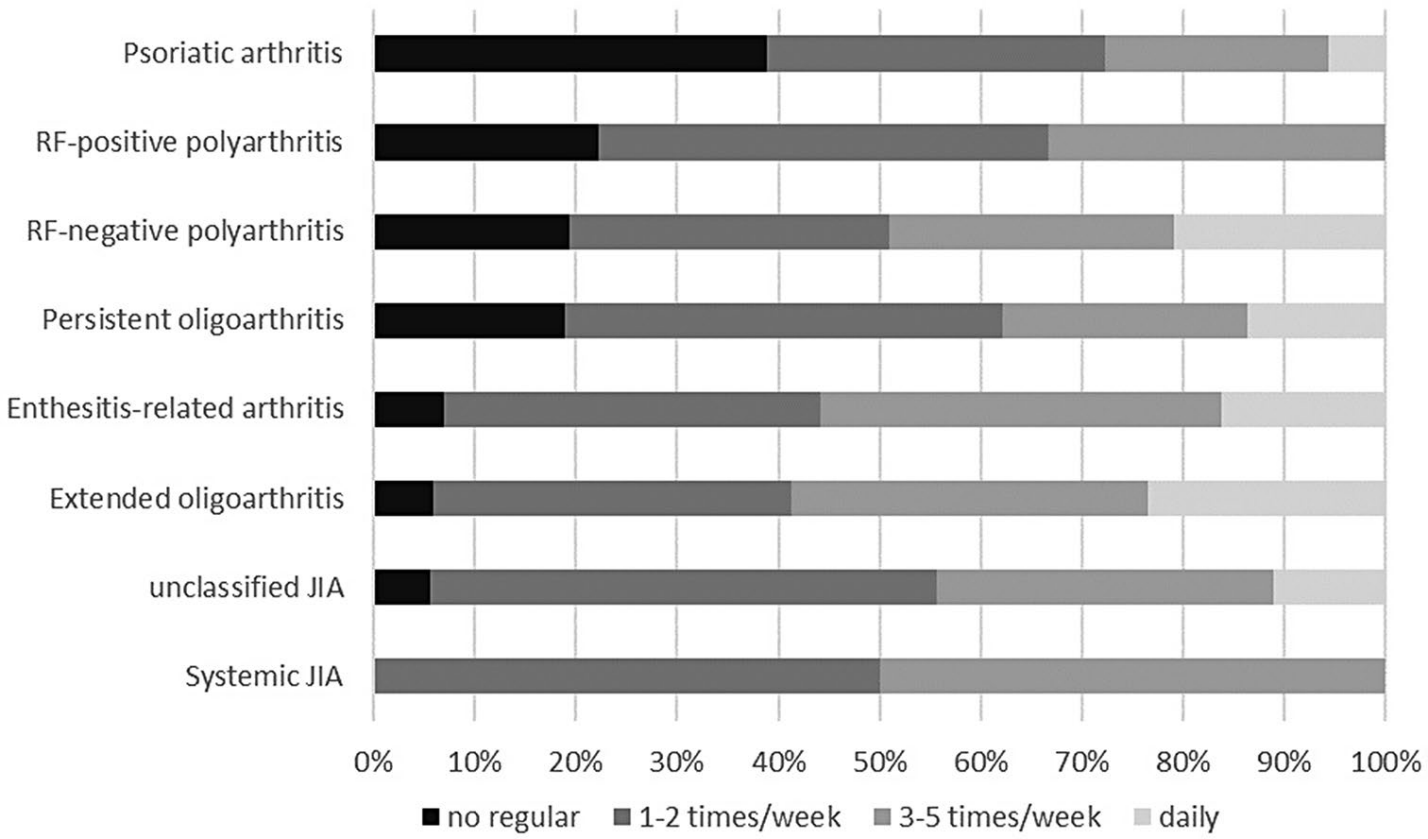
Total recreational screen time classified by JIA-category

Significant differences in mean total screen time were found between JIA categories at first documentation (omnibus test linear regression, *p* = 0.042), but not at 2-year follow-up (omnibus test linear regression, *p* = 0.235). Patients with systemic JIA and psoriatic arthritis showed longest mean screen times, while patients with persistent oligoarthritis reported the shortest (Fig. 2).

Multivariate associations between HRQoL, clinical/sociodemographic factors, and daily screen time are shown in Table 3. While low SES (OR 14.40; 95%-CI: 2.84–73.15) and increasing disease activity (OR 1.31; 95%-CI: 1.03–1.66) increased the likelihood of being assigned to group ≥ 4.5 h of daily screen time, better psychosocial health on the PedsQL (OR = 0.93, 95%-CI = 0.88–0.99) was associated with a lower likelihood of high daily screen time.

**Table 3:** Patient characteristics at 2-year follow-up categorized by daily total screen time

Variable	< 2 h/d	2-<3 h/d		3-<4.5 h/d		≥ 4.5 h/d	
No. of patients	53	39		55		67	
Female, no. (%)	39 (73.6)	29 (74.4)	OR 0.95; 95%CI: 0.26–3.42	31 (56.4)	OR 0.33; 95%CI: 0.11–1.05	36 (53.7)	OR 0.35; 95%CI: 0.11–1.11
BMI, mean ± SD	20.8 ± 2.7	21.7 ± 4.4	OR 1.04; 95%CI: 0.88–1.22	21.4 ± 3.0	OR 1.05; 95%CI: 0.90–1.23	22.3 ± 4.8	OR 1.08; 95%CI: 0.94–1.24
Low SES	18 (34.0)	17 (44.7)	OR 1.99; 95%CI: 0.47–8.47	23 (43.4)	OR 1.44; 95%CI: 0.37–5.52	**43 (65.2)**	**OR 14.40;** **95%CI: 2.84–73.15**
Medium SES	19 (35.8)	13 (34.2)	OR 0.63; 95%CI: 0.14–2.74	20 (37.7)	OR 1.01; 95%CI: 0.27–3.73	18 (27.3)	OR 4.59; 95%CI: 0.91–23.24
High SES	16 (30.2)	8 (21.1)	-	10 (18.9)	-	5 (7.6)	-
Disease duration, years, mean ± SD	4.7 ± 1.8	4.9 ± 1.8	OR 1.00; 95%CI: 0.74–1.36	4.4 ± 1.7	OR 0.92; 95%CI: 0.69–1.23	4.7 ± 2.5	OR 1.05; 95%CI: 0.81–1.37
cJADAS-10 score, mean ± SD	2.0 ± 2.1	3.1 ± 3.7	OR 1.27; 95%CI: 0.98–1.64	**3.6 ± 4.2**	**OR 1.31;** **95%CI: 1.03–1.66**	3.7 ± 4.5	OR 1.16; 95%CI: 0.92–1.47
PedsQL physical health, mean ± SD	87.6 ± 16.2	90.6 ± 13.0	OR 1.02; 95%CI: 0.96–1.08	90.1 ± 16.3	OR 1.06; 95%CI: 1.00-1.12	84.9 ± 20.4	OR 1.03; 95%CI: 0.98–1.08
PedsQL psychosocial health, mean ± SD	91.6 ± 10.7	94.2 ± 7.8	OR 1.00; 95%CI: 0.93–1.08	90.4 ± 14.0	OR 0.95; 95%CI: 0.90–1.02	**87.2 ± 14.6**	**OR 0.93;** **95%CI: 0.88–0.99**
No PA	3 (6.0)	2 (5.3)	OR 14.56; 95%CI: 0.70-302.49	2 (3.6)	OR 5.28; 95%CI: 0.21-132.53	6 (9.1)	OR 15.44; 95%CI: 0.93-256.51
< 1 time per week PA, no. (%)	3 (6.0)	7 (18.4)	OR 7.67; 95%CI: 0.81–72.83	3 (5.5)	OR 1.77; 95%CI: 0.16–19.06	7 (10.6)	OR 3.20; 95%CI: 0.40-25.28
1–2 times per week PA, no. (%)	19 (38.0)	9 (23.7)	OR 1.36; 95%CI: 0.17–10.72	23 (41.8)	OR 3.19; 95%CI: 0.59–17.06	29 (43.9)	OR 4.02; 95%CI: 0.84–19.14
3–5 times per week PA, no. (%)	14 (28.0)	14 (36.8)	OR 4.93; 95%CI: 0.70-34.59	20 (36.4)	OR 2.98; 95%CI: 0.54–16.45	17 (25.8)	OR 1.93; 95%CI: 0.36–10.20
Daily PA, no. (%)	11 (22.0)	6 (15.8)	-	7 (12.7)	-	7 (10.6)	**-**

< 2 h/day = reference

Missing values: SES *n* = 4 (1.8%); cJADAS-10 score *n* = 28 (13.1%); PedsQL *n* = 7 (3.3%), PA *n* = 5 (2.3%). Complete information was available for gender and disease duration

As shown in Table 4, female gender (OR = 0.12, 95% CI = 0.02–0.59) decreased the likelihood of being physically active daily, while better physical functioning on the PedsQL (OR = 1.08, 95% CI = 1.02–1.15) and daily SBM use less than 2 h increased the likelihood.

**Table 4:** Patient characteristics at 2-year follow-up categorized by weekly frequency of physical activity

Variable	Not regular	1–2 times per week		3–5 times per week		daily	
No. of patients	33	80		65		31	
Female, no. (%)	24 (72.7)	55 (68.8)	OR 0.84; 95%CI: 0.25–2.77	35 (53.8)	OR 0.68; 95%CI: 0.21–2.28	**18 (58.1)**	**OR 0.12;** **95%CI: 0.02–0.59**
BMI, mean ± SD	21.7 ± 4.9	21.5 ± 3.9	OR 1.00; 95%CI: 0.88–1.13	21.6 ± 3.1	OR 1.03; 95%CI: 0.90–1.16	21.5 ± 4.1	OR 0.95; 95%CI: 0.80–1.13
Low SES	16 (50.0)	38 (48.7)	OR 0.64; 95%CI: 0.14–2.91	28 (43.8)	OR 0.71; 95%CI: 0.15–3.40	16 (51.6)	OR 2.37; 95%CI: 0.32–17.42
Medium SES	11 (34.4)	26 (33.3)	OR 0.47; 95%CI: 0.10–2.14	22 (34.4)	OR 0.84; 95%CI: 0.18–4.02	11 (35.5)	OR 1.14; 95%CI: 1.48–8.81
High SES	5 (15.6)	14 (17.9)	-	14 (21.9)	**-**	4 (12.9)	-
Disease duration, years, mean ± SD	4.5 ± 2.6	4.8 ± 1.8	OR 0.98; 95%CI: 0.77–1.24	4.6 ± 1.7	OR 1.02; 95%CI: 0.79–1.33	4.8 ± 2.4	OR 0.91; 95%CI: 0.67–1.24
cJADAS-10 score, mean ± SD	3.71 ± 3.7	3.0 ± 3.8	OR 1.06; 95%CI: 0.88–1.28	2.7 ± 3.7	OR 1.07; 95%CI: 0.88–1.30	3.4 ± 4.4	OR 1.02; 95%CI: 0.81–1.28
PedsQL physical health, mean ± SD	82.5 ± 16.6	87.6 ± 18.1	OR 1.03; 95%CI: 0.98–1.08	93.8 ± 10.8	**OR 1.08;** **95%CI: 1.02–1.15**	85.9 ± 19.3	OR 0.98; 95%CI: 0.92–1.03
PedsQL psychosocial health, mean ± SD	86.4 ± 14.0	89.9 ± 12.4	OR 0.98; 95%CI: 0.93–1.04	93.9 ± 10.4	OR 0.97; 95%CI: 0.91–1.03	90.5 ± 14.6	OR 1.00; 95%CI: 0.93–1.07
SBM < 2 h/d, no. (%)	6 (18.2)	19 (23.8)	OR 1.43; 95%CI: 0.32–6.42	14 (21.5)	OR 3.13; 95%CI: 0.63–15.6	**11 (35.5)**	**OR 7.26;** **95%CI: 1.12–46.9**
SBM 2-<3 h/d, no. (%)	9 (27.3)	9 (11.3)	**OR 0.21;** **95%CI: 0.05–0.93**	14 (21.5)	OR 1.47; 95%CI: 0.37–5.86	6 (19.4)	OR 0.56; 95%CI: 0.07–4.64
SBM 3-<4.5 h/d, no. (%)	5 (15.2)	23 (28.8)	OR 1.78; 95%CI: 0.39–8.12	20 (30.8)	OR 3.61; 95%CI: 0.72–18.10	7 (22.6)	OR 2.99; 95%CI: 0.42–21.24
SBM ≥ 4.5 h/d, no. (%)	13 (39.4)	29 (36.3)	-	17 (26.2)	-	7 (22.6)	-

PA frequency (not regular = reference)

Missing values: SES *n* = 4 (1.9%); cJADAS-10 score *n* = 25 (11.9%); PedsQL *n* = 6 (2.89%). Complete information was available for gender, disease duration and SBM

### Comparison of PA level and SBM use between patients and peers over time

Characteristics at first documentation presented in Table 1 did not differ between patient and peer groups in terms of age and BMI. In contrast, significant differences were found for gender, SES, HRQoL, overall well-being, as well as overall pain.

At first documentation, 19.8% of patients and 4.3% of peers were identified as being physically active to the point of getting out of breath or sweating at most twice a month (Table 5). Overall, patients’ PA levels differed significantly from those of peers, with patients being less physically active on average (*p* = 0.004).

**Table 5:** Physical activity and total recreational screen time among adolescents with JIA and their peers, adjusted for SES and gender

	First documentation			2-year follow-up		
	JIA	Peers	*p-value*	JIA	Peers	*p-value*
**Physical activity level**			**0.004**			0.091
Nearly every day, no. (%)	40 (18.9)	30 (21.6)		31 (14.8)	24 (17.1)	
3–5 times/week, no. (%)	49 (23.1)	49 (35.3)		65 (31.1)	57 (40.7)	
1–2 times/week, no. (%)	81 (38.2)	54 (38.8)		80 (38.3)	46 (32.9)	
1–2 times/month, no. (%)	23 (10.8)	6 (4.3)		20 (9.6)	9 (6.4)	
Never, no. (%)	19 (9.0)	0 (0.0)		13 (6.2)	4 (2.9)	
**Screen time level**			0.447			0.681
< 2h/day, no. (%)	42 (21.0)	25 (18.4)		49 (24.5)	22 (16.7)	
2-2.5h/day, no. (%)	52 (26.0)	43 (31.6)		35 (17.5)	38 (28.8)	
3-4h/day, no (%)	47 (23.5)	40 (29.4)		52 (26.0)	43 (32.6)	
> 4h/day, no. (%)	59 (29.5)	28 (20.6)		64 (32.0)	29 (22.0)	
Total screen time^¥^, h/day	3.5 ± 2.6	3.0 ± 1.9	0.556	3.6 ± 2.5	3.3 ± 2.3	0.969

*JIA* juvenile idiopathic arthritis; *SES* socioeconomic status. ^**¥**^summed up across the media television/videos, computer/Internet, and gaming consoles

The proportion of JIA patients without regular PA (at most twice/month) decreased slightly over time, while the proportion of peers without regular PA simultaneously increased (OR 3.69; 95%CI: 1.01–13.50, *p* = 0.048). During the same period, PedsQl scores, well-being and pain levels in both groups converged. No group differences in overall PA levels were found at 2-year follow-up.

Significant gender-specific differences in overall PA level between the groups were found only at first documentation (Table 2). In fact, girls with JIA were significantly less physically active than girls without JIA (*p* < 0.001).

As shown in Table 5, mean total screen time of patients was higher than that of peers, but did not differ significantly at first documentation (patients 3.5 h vs. peers 3.0 h, *p* = 0.556) nor at 2-year follow-up (patients 3.6 h vs. 3.3 h, *p* = 0.969) when adjusting for SES and gender. Furthermore, no gender-specific differences were found between patient and peer groups in terms of total screen time, either at baseline or at follow-up (Table 2). The proportion of boys with JIA who consumed SBM for less than two hours per day increased over time from 14.1 to 17.4%, while the proportion of male peers with low SBM decreased from 17.7 to 10.0% (Table 2). However, total screen time remained constant in both groups during the observation period. The distribution of daily time spent with different screen media by both JIA patients and peers is shown in Table S1.

## Discussion

This study extends the current literature by providing information on PA levels and recreational SBM use in adolescents with JIA over time, taking into account cross-sectional clinical and sociodemographic correlates.

Results indicate that JIA patients with shorter disease duration were more physically inactive than their peers, but catch up to the level of their peers over the course of 24 months. Simultaneously, disease activity decreased and HRQoL increased. The amount of time spent using recreational SBM was stable over the 24-month course of the disease and comparable to that of their peers. Female patients reported spending less time on screens than male patients during the observation period, but were considered to be more physically inactive. Low SES, lower psychosocial health and higher disease activity were associated with a higher likelihood of heavy recreational SBM use. In addition, SBM use of less than 2 h per day and better physical health increased the likelihood of more frequent physical activity.

To date, studies on PA levels in adolescents with JIA are few and provide varying results. Our results are in line with recent reports showing that young people with JIA have lower PA levels than healthy controls [8, 42]. Other studies concerning PA of JIA patients diagnosed in the era of biologics found similar levels compared to controls [9, 27]. However, it should be noted that the comparability of previous studies is limited in some cases due to small sample sizes, narrow age ranges, underrepresentation of certain JIA categories, and differences in study populations’ disease state. In particular, distribution of JIA categories in the present study may be slightly skewed towards more severe disease. In addition, other characteristics not recorded in our or previous study populations, such as socioeconomic status or cultural characteristics, may have influenced the results.

In our study, PA levels increased slightly over the course of the disease, while in peers PA levels decreased over time. Declining levels of PA during adolescence are common and have been widely documented in the German general population [43]. Parallel to the observed increase in PA levels in our patients over time, a decrease in disease activity and pain intensity, an improvement in subjective physical health, and an increase in the proportion of patients receiving biologics therapy were registered. In similar longitudinal observations by Nordal et al. [44] decreasing disease activity was associated with increased participation in school sports.

As observed in our peer group, male patients were found to be more physically active than female patients. Similar gender-specific differences in PA levels have been found in adolescents with JIA in a previous study by Bohr et al. [42]. The results of our study on differences between male and female peers are also consistent with previous work showing that boys in the general population are more likely to adhere to PA guidelines than girls [45].

We did not find statistically significant differences in overall PA levels between JIA categories, but the proportion of adolescents without regular PA was higher in those with rheumatoid factor positive polyarthritis and psoriatic arthritis than in those with oligoarthritis. This could be explained on the one hand by a higher disease burden due to a larger number of active joints and on the other hand by the involvement of the axial skeleton with increasing pain during all weight-bearing activities, at least in some patients. In addition, the onset of disease in rheumatoid factor-positive polyarthritis often occurs during puberty, an age phase commonly associated with lower PA levels even in healthy individuals.

Being more physically active has been suggested as one way to enhance HRQoL and well-being among healthy adolescents and adults [46]. As our study design does not allow conclusions on causality of the association found between PA and HRQoL, future research designs should include RCTs involving interventions testing different modes and intensities of PA to characterize the effects of different forms of PA on aspects of HRQoL and well-being.

After adjusting for gender and SES, we found no statistically significant difference in overall recreational screen time between patients with JIA and their peers. Thus, our results are consistent with those of Sherman et al. [27], who also found no significant differences between patient and peer groups in a monocentric study.

Although there is no consensus on the amount of recommended screen time in children and adolescents, several experts have established guidelines advising them to limit recreational screen time to no more than 2 h a day [47]. Accordingly, almost 80% of JIA patients and more than 80% of peers exceeded the advised screen time. While increased screen time has frequently been linked to poorer health outcomes [48], there has recently been increasing debate about the degree to which the effects of screen time are also related to their content or even the context in which screens are used [49]. As certain content and contexts are thought to have potential benefits for individuals’ development, supporting young people with JIA to develop safer screen behaviors could also be an opportunity to promote social interactions and improve access to health information [49, 50].

In our study, low SES, lower psychosocial health, and higher disease activity were independently associated with a higher likelihood of extensive recreational SBM use. No studies in the area of JIA are known to date, but Ussher et al. [51] found comparable associations among adolescents without JIA. According to their results, increased use of television, videos and computers was linked to lower mental well-being. Another previous study of adolescents from the German general population showed that those with higher SES were more likely to meet the national screen time guidelines than those with lower SES [45].

The rising screen time with increasing disease activity in our study is consistent with previous studies [52, 53]. Children and adolescents with JIA are at greater risk for adopting a more sedentary lifestyle compared to their healthy peers in part due to disease related factors such as pain, fatigue, swollen and stiff joints. Billings et al. [54] further showed that social engagement in activities varied according to disease status. The more severe the disease activity the less patients participated in activities with friends and family members.

Furthermore, our study revealed that SBM use of less than 2 h per day increases the likelihood of more frequent PA. As screen media devices are often used while being sedentary, it seems likely that time displacement could be a primary mechanism through which SBM use may affect PA. However, recreational SBM use may also simply replace time otherwise spent being sedentary. Although previous studies examining the relationship between time spent on SBM and PA presented conflicting results, Pedersen et al. [55] recently showed that limiting recreational SBM use substantially increased children’s PA. High levels of recreational SBM use therefore emphasize the importance of developing and implementing measures to balance recreational SBM use to prevent physical inactivity.

In our study, male patients displayed significantly higher total screen time than female patients both at first documentation and at follow-up and were also more likely to be heavy users (> 4 h/day). Comparable gender-specific observations were also found in the peer group, but not in a recent survey by Hansen et al. [45] among over 15,000 German students in grades 5–10. However, Cavallo et al. [56] identified socio-demographic factors such as gender as potential determinants of leisure participation in children and adolescents with JIA.

With regard to JIA categories, differences in mean total screen time were only recorded at first documentation, with patients with systemic JIA presenting longest screen times. As systemic JIA was associated with lower physical leisure activity in a previous study [56], our result may reflect a higher disease burden in those affected. However, other (non-disease-related) factors not examined in our study may have contributed to higher use of recreational SBM in systemic JIA.

### Strengths and limitations

Some strengths of our study should be highlighted. In contrast to previous studies, we considered both PA and SBM as different, but not opposing aspects of physical inactivity during the course of the disease and under consideration of HRQoL subdomains. Other strengths include the multicenter design, large sample size, and the consideration of extensive sociodemographic and clinical data. This also included the consideration of SES, which is a well-known factor influencing adolescent health behaviour.

There are, however, also some limitations that have to be taken into account. First, although we were able to consider many associations with various variables, we do not have information on all potential determinants of PA and SBM, such as fatigue, pain, mental health, cultural background, or ethnicity. Furthermore, we are not able to provide information about the countries of origin, but assume that they were mainly in Europe. Concerning PA, it was argued not only that PA could increase HRQoL but also that better HRQoL could motivate further PA. Therefore, the associations may well be reciprocal and causation cannot be drawn. The same could be true for SBM. Second, both PA and SBM were self-reported by adolescents in our study. It is well recognized that the accuracy of self-reports is limited compared to objectively measurements. In particular, PA level might be over reported due to social desirability, inaccuracies may also occur from cognitive problems in recalling PA behaviour or in misunderstanding of the underlying concepts of the questions. However, most PA self-report measures, including single-items, are at least suitable for classifying subjects according to their PA levels [57, 58]. A measure similar to the one we used performed best compared to other short measures that can be used in large epidemiologic surveys [59, 60]. The use of more accurate objective measures (e.g. accelerometers) is preferable, but often not feasible in observational, longitudinal, multicenter studies such as the ICON.

The mere distinction between usage and non-usage of electronic media, the lacking assessment of media content and consideration of more devices (e.g. tablets/smartphones) are further limitations of this study. Future studies might try to apply more detailed (e.g. the distinction between week and weekend, and between offline and online usage) and more objective measures of media use. Finally, statements about PA and SBM use relate exclusively to the out-of-school context. It is therefore not possible to draw conclusions about time spent in front of screens for educational purposes and in physical education classes, which may have other impacts on individuals’ health.

## Conclusions

The present results indicate the heterogeneity of sedentary behaviours of adolescents with JIA, which differ less overall from those of their peers, but rather between males and females and as a function of SES. Accordingly, PA levels of JIA patients approach those of their peers and do not differ significantly in terms of screen time after adjusting for SES and gender. Females consume less recreational SBM but are considered more physically inactive than males. Although all forms of sedentary behaviour may pose health risks, recreational screen use may be particularly harmful as it has been associated with impaired psychosocial health, a common condition in chronically ill populations. Moreover, recreational SBM use of less than 2 h per day and better physical health increased the likelihood of more frequent PA.

Future research on health behaviours in young people with JIA should necessarily consider SES and gender in addition to clinical parameters in order to develop targeted interventions to promote healthy lifestyles for most vulnerable groups. In addition, qualitative studies are needed to explore patients’ perspectives on barriers, facilitators and beliefs regarding their recreational screen time and PA.

To benefit from the potential effects of reduced recreational screen use and regular PA on individuals’ health, pediatric rheumatologists, pediatricians and other healthcare professionals should educate patients and their relatives about the expected health-promoting effects. Behaviour change techniques including goal setting, motivational interviewing and supportive counseling can help to maintain desired health behaviours over time.

## Electronic supplementary material

Below is the link to the electronic supplementary material.

Supplementary Material 1

## Data Availability

The datasets used and/or analysed during the current study are available from the corresponding author on reasonable request.

## Abbreviations

CHAQ: Childhood Health Assessment Questionnaire
cJADAS10: Clinical Juvenile Arthritis Disease Activity Score in 10 joints
DMARDs: Disease Modifying antirheumatic drugs
GC: Glucocorticoid
HRQoL: Health-related quality of life
ICON: Inception Cohort of Newly diagnosed patients with JIA
ILAR: International League of Associations for Rheumatology
JIA: Juvenile idiopathic arthritis
NRS: Numerical Rating Scale
NSAID: Non-steroidal anti-inflammatory drugs
PA: Physical activity
PedsQL: Pediatric Quality of Life Inventory
RCT: Randomized Controlled Trial
SBM: Screen-based media
SES: Socioeconomic status
WHO: World Health Organization

## References

[1] Ravelli A, Martini A. Juvenile idiopathic arthritis. Lancet. 2007;369:767–78. 17336654 10.1016/S0140-6736(07)60363-8

[2] Rashid A, Cordingley L, Carrasco R, et al. Patterns of pain over time among children with juvenile idiopathic arthritis. Arch Dis Child. 2018;103:437–43. 29175824 10.1136/archdischild-2017-313337 PMC5916104

[3] Hutzal CE, Wright FV, Stephens S, Schneiderman-Walker J, Feldman BM. A qualitative study of fitness instructors’ experiences leading an exercise program for children with juvenile idiopathic arthritis. Phys Occup Ther Pediatr. 2009;29:409–25. 19916825 10.3109/01942630903245309

[4] Tong A, Jones J, Craig JC, Singh-Grewal D. Children’s experiences of living with juvenile idiopathic arthritis: a thematic synthesis of qualitative studies. Arthritis Care Res. 2012;64:1392–404. 10.1002/acr.21695 22504867

[5] Lundberg V, Eriksson C. Health-related quality of life among Swedish children with juvenile idiopathic arthritis: parent-child discrepancies, gender differences and comparison with a European cohort. Pediatr Rheumatol Online J. 2017;15:26. 28403864 10.1186/s12969-017-0153-5 PMC5389151

[6] Bechtold S, Simon D. Growth abnormalities in children and adolescents with juvenile idiopathic arthritis. Rheumatol Int. 2014;34:1483–8. 24760485 10.1007/s00296-014-3022-2

[7] Räsänen K, Markula-Patjas K, Kantanen S, Sipilä K, Lakka TA, Arikoski P, Piippo-Savolainen E. Impaired cardiorespiratory and neuromuscular fitness in children and adolescents with juvenile idiopathic arthritis: a cross-sectional case-control study in the era of biologic drug therapies. Pediatr Rheumatol Online J. 2023;21:26. 36932386 10.1186/s12969-023-00808-9 PMC10022213

[8] Lelieveld OT, Armbrust W, van Leeuwen MA, Duppen N, Geertzen JH, Sauer PJ, van Weert E. Physical activity in adolescents with juvenile idiopathic arthritis. Arthritis Rheum. 2008;59:1379–84. 18821655 10.1002/art.24102

[9] Risum K, Hansen BH, Selvaag AM, Molberg Ø, Dagfinrud H, Sanner H. Physical activity in patients with oligo- and polyarticular juvenile idiopathic arthritis diagnosed in the era of biologics: a controlled cross-sectional study. Pediatr Rheumatol Online J. 2018;16:64. 30333025 10.1186/s12969-018-0281-6 PMC6192283

[10] Saidi O, Rochette E, Bourdier P, Ratel S, Merlin E, Pereira B, et al. Sleep in children and adolescents with juvenile idiopathic arthritis: a systematic review and meta-analysis of case-control studies. Sleep. 2022;45:zsab233. 34525202 10.1093/sleep/zsab233

[11] Grönlund MM, Kaartoaho M, Putto-Laurila A, Laitinen K. Juvenile idiopathic arthritis patients with low inflammatory activity have increased adiposity. Scand J Rheumatol. 2014;43:488–92. 25178152 10.3109/03009742.2014.918171

[12] Lee IM, Shiroma EJ, Lobelo F, Puska P, Blair SN, Katzmarzyk PT. Effect of physical inactivity on major non-communicable diseases worldwide: an analysis of burden of disease and life expectancy. Lancet. 2012;380:219–29. 22818936 10.1016/S0140-6736(12)61031-9 PMC3645500

[13] Proper KI, Singh AS, van Mechelen W, Chinapaw MJ. Sedentary behaviors and health outcomes among adults: a systematic review of prospective studies. Am J Prev Med. 2011;40:174–82. 21238866 10.1016/j.amepre.2010.10.015

[14] for the Committee on Adolescent Health Care Services. And models of care for treatment, Prevention, and Healthy Development. Adolescent health services: missing opportunities. Washington, DC: National Academies; 2008.

[15] Domingues-Montanari S. Clinical and psychological effects of excessive screen time on children. J Paediatr Child Health. 2017;53:3338. 10.1111/jpc.13462 28168778

[16] Stiglic N, Viner RM. Effects of screentime on the health and well-being of children and adolescents: a systematic review of reviews. BMJ Open. 2019;9:e023191. 30606703 10.1136/bmjopen-2018-023191 PMC6326346

[17] Pagani LS, Lévesque-Seck F, Fitzpatrick C. Prospective associations between televiewing at toddlerhood and later self-reported social impairment at middle school in a Canadian longitudinal cohort born in 1997/1998. Psychol Med. 2016;46:3329–37. 27618949 10.1017/S0033291716001689

[18] Twenge JM, Campbell WK. Associations between screen time and lower psychological well-being among children and adolescents: evidence from a population-based study. Prev Med Rep. 2018;12:271–83. 30406005 10.1016/j.pmedr.2018.10.003 PMC6214874

[19] Wu XY, Han LH, Zhang JH, Luo S, Hu JW, Sun K. The influence of physical activity, sedentary behavior on health-related quality of life among the general population of children and adolescents: a systematic review. PLoS ONE. 2017;12:e0187668. 29121640 10.1371/journal.pone.0187668 PMC5679623

[20] Clark SL, Denburg MR, Furth SL. Physical activity and screen time in adolescents in the chronic kidney disease in children (CKiD) cohort. Pediatr Nephrol. 2016;31:801–8. 26684326 10.1007/s00467-015-3287-z PMC4924924

[21] Walker RG, Obeid J, Nguyen T, Ploeger H, Proudfoot NA, Bos C, Chan AK, Pedder L, Issenman RM, Scheinemann K, Larché MJ, McAssey K, Timmons BW. Sedentary time and screen-based sedentary behaviors of children with a chronic disease. Pediatr Exerc Sci. 2015;27:219–25. 25389217 10.1123/pes.2014-0074

[22] Leszczak J, Weres A, Baran J, Wyszyńska J, Grzegorczyk J, Lewandowski B, Mazur A. Sedentary behaviors in children and adolescents with type 1 diabetes, depending on the insulin therapy used. Med (Baltim). 2019;98:e15625. 10.1097/MD.0000000000015625 PMC6531069 31083260

[23] Elmesmari R, Reilly JJ, Martin A, Paton JY. Accelerometer measured levels of moderate-to-vigorous intensity physical activity and sedentary time in children and adolescents with chronic disease: a systematic review and meta-analysis. PLoS ONE. 2017;12:e0179429. 28640907 10.1371/journal.pone.0179429 PMC5480890

[24] Pinto AJ, Roschel H, de Sá Pinto AL, Lima FR, Pereira RMR, Silva CA, Bonfá E, Gualano B. Physical inactivity and sedentary behavior: overlooked risk factors in autoimmune rheumatic diseases? Autoimmun Rev. 2017;16:667–74. 28479487 10.1016/j.autrev.2017.05.001

[25] Gualano B, Bonfa E, Pereira RMR, Silva CA. Physical activity for paediatric rheumatic diseases: standing up against old paradigms. Nat Rev Rheumatol. 2017;13:368–79. 28533552 10.1038/nrrheum.2017.75

[26] Bourdier P, Saidi O, Rochette E, Ratel S, Merlin E, Pereira B, Duché P. Physical activity and sedentary levels in children with juvenile idiopathic arthritis and inflammatory bowel disease. A systematic review and meta-analysis. Pediatr Res. 2019;86:149–56. 31029060 10.1038/s41390-019-0409-5

[27] Sherman G, Nemet D, Moshe V, Consolaro A, Ravelli A, Ruperto N, Uziel Y. Disease activity, overweight, physical activity and screen time in a cohort of patients with juvenile idiopathic arthritis. Clin Exp Rheumatol. 2018;36:1110–16. 29600947

[28] Sengler C, Klotsche J, Niewerth M, Liedmann I, Foll D, Heiligenhaus A, Ganser G, Horneff G, Haas JP, Minden K. The majority of newly diagnosed patients with juvenile idiopathic arthritis reach an inactive disease state within the first year of specialised care: data from a German inception cohort. RMD Open. 2015;1:e000074. 26688748 10.1136/rmdopen-2015-000074 PMC4680591

[29] Petty RE, Southwood TR, Manners P, Baum J, Glass DN, Goldenberg J, He X, Maldonado-Cocco J, Orozco-Alcala J, Prieur AM, et al. International League of Associations for Rheumatology classification of juvenile idiopathic arthritis: second revision, Edmonton, 2001. J Rheumatol. 2004;31:390–2. 14760812

[30] Kromeyer-Hauschild K, Moss A, Wabitsch M. Referenzwerte für den body-Mass-Index für Kinder, Jugendliche Und Erwachsene in Deutschland: Anpassung Der AGA-BMI_Referenz Im Altersbereich Von 15 bis 18. Jahren Adipositas. 2015;9:123–7.

[31] Kromeyer-Hauschild K, Wabitsch M, Kunze D, Geller F, Geiß HC, Hesse V, et al. Perzentile für den body-mass-index für das Kindes- Und Jugendalter Unter Heranziehung verschiedener deutscher Stichproben. Monatsschrift Kinderheilkunde. 2001;149:807–18.

[32] Foeldvari I, Ruperto N, Dressler F, Hafner R, Kuster RM, Michels H, et al. The German version of the childhood health assessment questionnaire (CHAQ) and the child health questionnaire (CHQ). Clin Exp Rheumatol. 2001;19(Suppl 23):S71–5. 11510335

[33] Consolaro A, Negro G, Chiara Gallo M, Bracciolini G, Ferrari C, Schiappapietra B, et al. Defining criteria for disease activity states in nonsystemic juvenile idiopathic arthritis based on a three-variable juvenile arthritis disease activity score. Arthritis Care Res (Hoboken). 2014;66:1703–9. 24980508 10.1002/acr.22393

[34] Varni JW, Seid M, Smith Knight T, Burwinkle T, Brown J, Szer IS. The PedsQL in pediatric rheumatology: reliability, validity, and responsiveness of the Pediatric Quality of Life Inventory Generic Core Scales and Rheumatology Module. Arthritis Rheum. 2002;46:714–25. 11920407 10.1002/art.10095

[35] Varni JW, Burwinkle TM, Seid M, Skarr D. The PedsQL 4.0 as a pediatric population health measure: feasibility, reliability, and validity. Ambul Pediatr. 2003;3:329–41. 14616041 10.1367/1539-4409(2003)003<0329:tpaapp>2.0.co;2

[36] Lampert T, Muters S, Stolzenberg H, Kroll LE. Measurement of socioeconomic status in the KiGGS study: first follow-up (KiGGS Wave 1). Bundesgesundheitsblatt Gesundheitsforschung Gesundheitsschutz. 2014;57:762–70. 24950825 10.1007/s00103-014-1974-8

[37] Listing M, Mönkemoller K, Liedmann I, Niewerth M, Sengler C, Listing J, et al. The majority of patients with newly diagnosed juvenile idiopathic arthritis achieve a health-related quality of life that is similar to that of healthy peers: results of the German multicenter inception cohort (ICON). Arthritis Res Ther. 2018;20:106. 29848349 10.1186/s13075-018-1588-x PMC5977761

[38] Lampert T, Sygusch R, Schlack R. Use of electronic media in adolescence. Results of the German health interview and examination survey for children and adolescents (KIGGS). Bundesgesundheitsblatt Gesundheitsforschung Gesundheitsschutz. 2007;50:643–52. 17514448 10.1007/s00103-007-0225-7

[39] Schmitz KH, Harnack L, Fulton JE, Jacobs DR Jr, Gao S, Lytle LA, Coevering PV. Reliability and validity of a brief questionnaire to assess television viewing and computer use. J Sch Health. 2004;74:370–7. 15656264 10.1111/j.1746-1561.2004.tb06632.x

[40] Utter J, Neumark-Sztainer D, Jeffery R, Story M. Couch potatoes or French fries: are sedentary behaviors associated with body mass index, physical activity, and dietary behaviors among adolescents? J Am Diet Assoc. 2003;103:1298–305. 14520247 10.1016/s0002-8223(03)01079-4

[41] Van Der Horst K, Paw MJ, Twisk JW, Van Mechelen W. A brief review on correlates of physical activity and sedentariness in youth. Med Sci Sport Exer. 2007;39:1241–50. 10.1249/mss.0b013e318059bf35 17762356

[42] Bohr AH, Nielsen S, Müller K, Karup Pedersen F, Andersen LB. Reduced physical activity in children and adolescents with juvenile idiopathic arthritis despite satisfactory control of inflammation. Pediatr Rheumatol Online J. 2015;13:57. 26653716 10.1186/s12969-015-0053-5 PMC4676098

[43] Finger JD, Varnaccia G, Borrmann A, Lange C, Mensink GBM. Physical activity among children and adolescents in Germany. Results of the cross-sectional KiGGS Wave 2 study and trends. J Health Monit. 2018;3:23–30. 35586180 10.17886/RKI-GBE-2018-023.2 PMC8848914

[44] Nordal E, Rypdal V, Arnstad ED, Aalto K, Berntson L, Ekelund M, et al. Participation in school and physical education in juvenile idiopathic arthritis in a nordic long-term cohort study. Pediatr Rheumatol Online J. 2019;17:44. 31307487 10.1186/s12969-019-0341-6 PMC6631827

[45] Hansen J, Hanewinkel R, Galimov A. Physical activity, screen time, and sleep: do German children and adolescents meet the movement guidelines? Eur J Pediatr. 2022;181:1985–95. 35113254 10.1007/s00431-022-04401-2 PMC8811591

[46] Marquez DX, Aguiñaga S, Vásquez PM, Conroy DE, Erickson KI, Hillman C, et al. A systematic review of physical activity and quality of life and well-being. Transl Behav Med. 2020;10:1098–109. 33044541 10.1093/tbm/ibz198 PMC7752999

[47] Tremblay MS, Carson V, Chaput JP, Gorber SC, Dinh T, Duggan M, et al. Canadian 24-Hour Movement Guidelines for Children and Youth: an integration of physical activity, sedentary Behaviour, and Sleep. Appl Physiol Nutr Metab. 2016;41(Suppl 3):S311–27. 27306437 10.1139/apnm-2016-0151

[48] Carson V, Hunter S, Kuzik N, Gray CE, Poitras VJ, Chaput JP, et al. Systematic review of sedentary behaviour and health indicators in school-aged children and youth: an update. Appl Physiol Nutr Metab. 2016;41(Suppl 3):S240–65. 27306432 10.1139/apnm-2015-0630

[49] Huang S, Lai X, Zhao X, Dai X, Yao Y, Zhang C, et al. Beyond screen time: exploring the associations between types of Smartphone Use Content and adolescents’ Social relationships. Int J Environ Res Public Health. 2022;19:8940. 35897307 10.3390/ijerph19158940 PMC9331893

[50] Miri S, Ferjani HL, Nessib DB, Majdoub F, Kaffel D, Maatallah K, et al. Growing up with juvenile idiopathic arthritis: social issues. Revista Colombiana de Reumatología; 2023.

[51] Ussher MH, Owen CG, Cook DG, Whincup PH. The relationship between physical activity, sedentary behaviour and psychological wellbeing among adolescents. Soc Psychiatry Psychiatr Epidemiol. 2007;42:851–6. 17639309 10.1007/s00127-007-0232-x

[52] Cassidy JT, Petty RE. 2005. Chronic arthritis in childhood. In: Cassidy JT, Petty RE, Laxer RM, Lindsley CB, editors. Textbook of pediatric rheumatology. pp.206–260.

[53] Schanberg LE, Anthony KK, Gil KM, Maurin EC. Daily pain and symptoms in children with polyarticular arthritis. Arthritis Rheum. 2003;48:1390–97. 12746912 10.1002/art.10986

[54] Billings AG, Moos RH, Miller JJ 3rd, Gottlieb JE. Psychosocial adaptation in juvenile rheumatic disease: a controlled evaluation. Health Psychol. 1987;6:343–59. 3608946 10.1037//0278-6133.6.4.343

[55] Pedersen J, Rasmussen MGB, Sørensen SO, Mortensen SR, Olesen LG, Brønd JC, et al. Effects of limiting recreational screen media use on physical activity and sleep in families with children: a Cluster Randomized Clinical Trial. JAMA Pediatr. 2022;176(8):741–9. 35604678 10.1001/jamapediatrics.2022.1519 PMC9127712

[56] Cavallo S, April KT, Grandpierre V, Majnemer A, Feldman DE. Leisure in children and adolescents with juvenile idiopathic arthritis: a systematic review. PLoS ONE. 2014;9:e104642. 25329390 10.1371/journal.pone.0104642 PMC4203655

[57] Mâsse LC, de Niet JE. Sources of validity evidence needed with self-report measures of physical activity. J Phys Act Health. 2012;9(Suppl 1):S44–55. 22287447 10.1123/jpah.9.s1.s44

[58] Milton K, Bull FC, Bauman A. Reliability and validity testing of a single-item physical activity measure. Br J Sports Med. 2011;45:203–8. 20484314 10.1136/bjsm.2009.068395

[59] Booth ML, Okely AD, Chey T, Bauman A. The reliability and validity of the physical activity questions in the WHO health behaviour in schoolchildren (HBSC) survey: a population study. Br J Sports Med. 2001;35:263–7. 11477024 10.1136/bjsm.35.4.263 PMC1724349

[60] Prochaska JJ, Sallis JF, Long B. A physical activity screening measure for use with adolescents in primary care. Arch Pediatr Adolesc Med. 2001;155:554–9. 11343497 10.1001/archpedi.155.5.554

