# Supplementary material for: Physical (in)activity and screen-based media use of adolescents with juvenile idiopathic arthritis over time - data from a German inception cohort
Source: Pediatr Rheumatol Online J. 2024 Oct 21;22:93. doi: 10.1186/s12969-024-01027-6 (PMC11492743; doi:10.1186/s12969-024-01027-6)
Supplement: Supplementary file 1 — Supplementary Material 1 [file 12969_2024_1027_MOESM1_ESM.docx]

**Table S1** Average amount of daily time spent with different screen media among adolescents with JIA and controls

| **Screen-based media** | **First documentation** | | **2-year follow-up** | |
| --- | --- | --- | --- | --- |
|  | JIA | controls | JIA | controls |
| **TV/videos**  At most 2h/day, no. (%)  At least 3h/day, no. (%) | 181 (85.8)  30 (14.2) | 123 (88.5)  16 (11.5) | 180 (86.5)  28 (13.5) | 129 (92.8)  10 (7.2) |
| **Computer/Internet**  At most 2h/day, no. (%)  At least 3h/day, no. (%) | 161 (77.0)  48 (23.0) | 114 (82.0)  25 (18.0) | 149 (72.3)  57 (27.7) | 101 (73.7)  36 (26.3) |
| **Gaming consoles**  At most 2h/day, no. (%)  At least 3h/day, no. (%)  **Mobile phone**  At most 2h/day, no. (%)  At least 3h/day, no. (%) | 192 (93.7)  13 (6.3)  146 (68.5)  67 (31.5) | 134 (97.8)  3 (2.2)  113 (80.1)  28 (19.9) | 190 (92.2)  16 (7.8)  114 (55.1)  93 (44.9) | 129 (96.3)  5 (3.7)  84 (60.4)  55 (39.6) |

JIA, juvenile idiopathic arthritis.
